# Semen CD4^+^ T Cells and Macrophages Are Productively Infected at All Stages of SIV infection in Macaques

**DOI:** 10.1371/journal.ppat.1003810

**Published:** 2013-12-12

**Authors:** Sibylle Bernard-Stoecklin, Céline Gommet, Aurélien B. Corneau, Sabrina Guenounou, Claire Torres, Nathalie Dejucq-Rainsford, Antonio Cosma, Nathalie Dereuddre-Bosquet, Roger Le Grand

**Affiliations:** 1 CEA, Division of Immuno-Virology, iMETI/DSV, Fontenay-aux-Roses, France; 2 UMR-E1, Université Paris Sud 11, Orsay, France; 3 Vaccine Research Institute (VRI), Créteil, France; 4 INSERM U1085-IRSET, Université de Rennes 1, Structure Fédérative Recherche Biosit, Campus de Beaulieu, Rennes, France; Emory University, United States of America

## Abstract

The mucosal events of HIV transmission have been extensively studied, but the role of infected cells present in the genital and rectal secretions, and in the semen, in particular, remains a matter of debate. As a prerequisite to a thorough *in vivo* investigation of the early transmission events through infected cells, we characterized in detail by multi-parameter flow cytometry the changes in macaque seminal leukocytes during SIVmac251 infection, focusing on T cells, macrophages and dendritic cells. Using immunocytofluorescence targeting SIV proteins and real-time quantitative PCR targeting SIV DNA, we investigated the nature of the infected cells on sorted semen leukocytes from macaques at different stages of infection. Finally, we cocultured semen CD4^+^ T cells and macrophages with a cell line permissive to SIV infection to assess their infectivity *in vitro*. We found that primary infection induced strong local inflammation, which was associated with an increase in the number of leukocytes in semen, both factors having the potential to favor cell-associated virus transmission. Semen CD4^+^ T cells and macrophages were productively infected at all stages of infection and were infectious *in vitro*. Lymphocytes had a mucosal phenotype and expressed activation (CD69 & HLA-DR) and migration (CCR5, CXCR4, LFA-1) markers. CD69 expression was increased in semen T cells by SIV infection, at all stages of infection. Macrophages predominated at all stages and expressed CD4, CCR5, MAC-1 and LFA-1. Altogether, we demonstrated that semen contains the two major SIV-target cells (CD4+ T cells and macrophages). Both cell types can be productively infected at all stages of SIV infection and are endowed with markers that may facilitate transmission of infection during sexual exposure.

## Author Summary

Human Immunodeficiency Virus infection is predominantly transmitted by mucosal exposure, after sexual intercourse. Although substantial progresses have been recently achieved in our understanding of the mechanisms of HIV mucosal transmission, many questions remain. Semen is one of the major sources for HIV which contains both cell-free viral particles and viral infected cells. However, today, the role of cell-associated virus has been largely understudied. We provide here a detailed characterization of the semen leukocyte populations in the highly relevant experiment model of SIV infection of macaques. We demonstrate that the major target cells for the virus, CD4^+^ T cells and macrophages, are present in macaques semen at all stages of infection. Both cell types are productively infected *in vivo* and are endowed with adhesion and migration markers that may facilitate virus transmission during sexual exposure. The acute phase of infection is associated with a strong seminal inflammation that may increase semen leukocytes infectivity. This work supports for a role of cell-associated virus in HIV transmission which needs to be considered for the design of prevention strategies.

## Introduction

More than 33 million people are currently living with HIV/AIDS worldwide. Almost 80% of new infections occur through sexual intercourse. Semen is thus one of the major factors in HIV transmission. Most studies on HIV sexual transmission have focused on the role of cell-free particles, and the underlying mechanisms of transmission have been extensively described. Moreover, most attempts to develop HIV vaccines and microbicides have focused on blocking cell-free virus transmission. The rectal and vaginal exposure of macaques to free SIV particles has been widely used in studies of the sexual transmission of HIV and evaluations of the efficacy of prophylactic strategies [1], [2]. Most challenge studies use viruses produced *in vitro* in the culture supernatants of human and nonhuman primate (NHP) cells.

However, genital secretions, including semen, contain HIV in both cell-free and cell-associated forms. The prevalence of proviral DNA in semen ranges from 21% to 65% in HIV-infected patients, and high levels of viral DNA have been associated with high leukocyte counts in semen [3]. Moreover, leukocytospermia, the incidence of which is higher in seropositive than in seronegative individuals [3], has been associated with a high degree of semen infectiousness [4]. This suggests that semen leukocytes may also be an important factor to be taken into account when considering the mucosal transmission of HIV. In the first few years of the HIV epidemic, the hypothesis that HIV could be efficiently transmitted by infected cells, through direct cell-to-cell contact, was proposed, as most retroviruses spread in this way [5].

It is now clearly established that HIV does indeed spread through cell contacts [6], [7]. Moreover, in reconstituted mucosal models, HIV-infected cells efficiently transmit the virus across epithelial barriers. If the epithelial surface is intact, viral translocation involves transcytosis, which is favored by viral synapses between productive cells and epithelial cells [8], [9], or direct uptake by local target cells, such as Langerhans cells, macrophages and intraepithelial T cells [10], [11]. We and others have demonstrated that the vaginal inoculation of humanized mice and macaques with infected leukocytes induces systemic infection. Inoculated CFSE-labeled cells were found in the vaginal tissue and the draining lymph nodes within 21 h, suggesting that these cells were able to migrate through the mucosal epithelium and to disseminate rapidly [12], [13].

We have reported that macrophages and CD4^+^ T cells present in the various secretory glands of the male NHP genital tract may efficiently seed the semen with free viral particles and infected cells [14]. However, semen from NHP is difficult to collect and process, so previous studies made use of spleen cells from infected animals (our reported work) or *ex vivo* infected PBMCs, as semen cell surrogates [12], [13], [15]. These cell-associated virus stocks may not be representative of leukocyte-infected populations in semen. Indeed, semen leukocytes, like mucosal cells, may have a distribution and differentiation and activation phenotypes different from those of blood and spleen cells. Therefore, it is of prime importance to thoroughly investigate the nature and characteristics of the potential cell transmitters in the semen of NHP, since this model is unique to decipher *in vivo* the early events of HIV sexual transmission.

Very few studies have focused on the nature and phenotype of the infected cells present in semen. There is still no formal proof that these cells can transmit HIV across mucosal barriers, and the mechanisms potentially involved have yet to be identified. An understanding of the contribution of semen leukocytes to the sexual transmission of HIV may have significant implications for prevention strategies.

We aimed to carry out a more detailed characterization of the infected cells present in semen, elucidating their role in mucosal transmission, in the experimental infection of macaques with pathogenic SIVmac251 model. We focused on characterization of the semen leukocyte populations, the nature of the infected cells and their capacity to transmit infection *in vitro*. We demonstrated that all major viral target cells, including CD4^+^ T cells and macrophages are productively infected at every stage of infection.

## Results

### Virus shedding in the semen of SIVmac251-infected macaques

**Figure 1:**
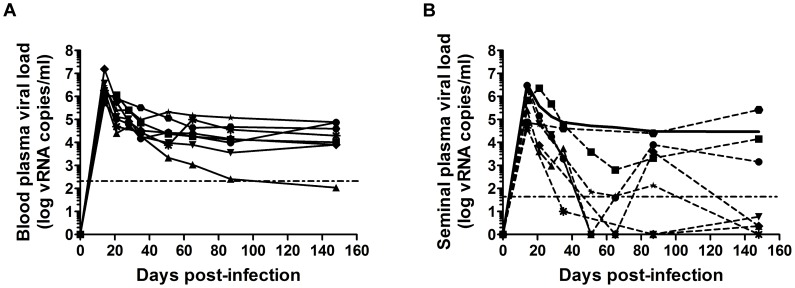
Viral shedding in blood and semen in macaques. (A–B) Longitudinal follow-up of RNA viral loads in blood (A) and seminal plasma (B) in 8 macaques infected intravenously with high doses of SIVmac251 (5,000 AID_50_); each animal is represented by different dot. The solid black line indicates the mean PVL. The dotted horizontal line represents the limit of quantification (111 and 37 copies/ml in blood and semen plasma respectively).

We assumed that, similarly to men, macaque semen contains various types of infectious material, including free viral particles and infected leukocytes, and that current challenge studies in NHP models of HIV and AIDS do not fully reproduce the conditions of natural transmission. We analyzed the dynamics of free virus shedding in macaque semen, in longitudinal and transverse studies in cynomolgus macaques infected with pathogenic SIVmac251. As in HIV-infected patients, we found a strong positive correlation between blood plasma (PVL) and seminal plasma (SVL) viral RNA (vRNA) loads (Spearman *r* = 0.6381, *p* = 0.0001, *n* = 30) [16], [17], [18], [19]. After the first month of infection, SVL is variable and correlated with PVL (Fig. 1 A–B). Mean SVL remained systematically equal to, or lower than PVL (Fig. 1 B). However, as observed in humans, discordant profiles are also observed.

### Changes in semen leukocytes in SIVmac251-infected macaques

We investigated whether semen leukocytes could be infected and play a role in the mucosal transmission of HIV/SIV, by carrying out a detailed characterization by multiple approaches. We first compared semen cells from uninfected and SIVmac251-infected cynomolgus macaques at various stages of infection.

**Figure 2:**
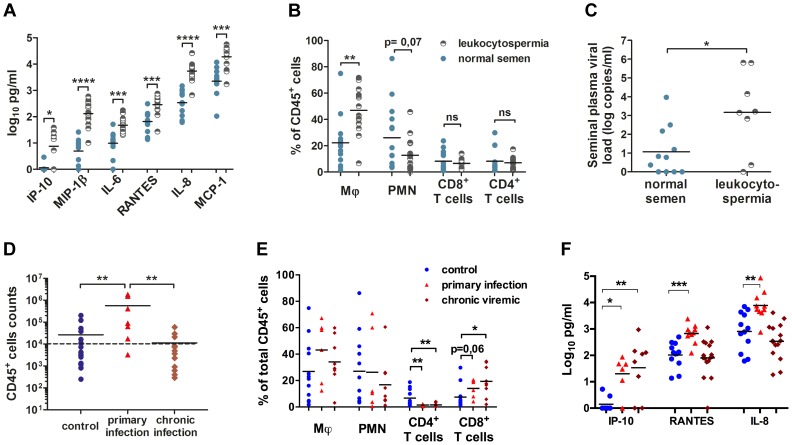
Semen leukocytes in macaques. (A) Seminal concentrations of six pro-inflammatory molecules in normal and leukocytospermic animals. (B) Proportion of each studied leukocyte subset among total CD45^+^ cells. (C) Semen vRNA load in SIV^+^ macaques with normal (*n* = 11, blue circle) or leukocytospermic (*n* = 8, half plein black circles) semen. (D) Number of CD45^+^ events acquired by flow cytometry per sample since the total collected semen cells were analyzed, for control uninfected (*n* = 20), and SIV-infected macaques, at 10 and 14 dpi (*n* = 7) and during chronic infection. The dotted line represents the leukocytospermia threshold (10,000 CD45^+^ events acquired) (*n* = 13). (E) Proportion of each subset among total CD45^+^ events in SIV^−^ macaques (*n* = 12, blue circle) and SIV^+^ macaques during primary infection (*n* = 7, red triangle) and chronic infection (*n* = 9, red diamond). (F) Seminal concentrations of IP-10, RANTES and IL-8 in SIV^−^ macaques (*n* = 8 for IP-10 and *n* = 12 for RANTES and IL-8, blue circle) and SIV^+^ macaques during primary infection (*n* = 6 for IP-10 and *n* = 10 for RANTES and IL-8, red triangle) and chronic infection (*n* = 8 for IP-10 and *n* = 16 for RANTES and IL-8, red diamond).

In uninfected animals, the semen contained various amounts of leukocytes, the numbers of which were strongly correlated with markers of inflammation (Fig. 2 A). Indeed, leukocytospermic individuals had significantly higher concentrations of inflammatory cytokines, including IP-10 (*p* = 0.0157), MIP1β (*p* = 0.0002), IL-6 (*p* = 0.0004), RANTES (*p* = 0.0007), IL-8 (*p* = 0.0007) and MCP-1 (*p* = 0.0089), in seminal plasma. The levels of all these molecules, except MIP-1β, were affected by SIV infection (Table S1). IP10, Il-8 and RANTES concentrations were significantly higher in the seminal plasma of macaques during primary infection than in uninfected macaques. The concentrations of IL-8, RANTES, IL-6 and MCP-1 were correlated with PVL and/or SVL.

At steady state, macaque semen contains mostly polymorphonuclear cells (PMN, CD45^+^CD11b^+^HLA-DR^−^, 26.02%±6.70% of total CD45^+^ cells), macrophages (CD45^+^CD3^−^CD8^−^CD11b^+^HLA-DR^+^, 22.22%±5.06% of total CD45^+^ cells) and T cells (8.15%±1.94% CD4^+^ T cells and 8.11%±2.36% CD8^+^ T cells; Fig. 2 B, Fig. S1). A small proportion of dendritic cells was also identified, corresponding to a mean of 1.87%±1.20% of total CD45^+^ cells (Fig. S1). Inflammation affected the proportions of macrophages and PMN, with a significant increase of the proportion of macrophages among total CD45^+^ cells (Mann-Whitney test, *p* = 0.0022) and a relative decrease in the proportion of PMN (Mann-Whitney test, *p* = 0.0769; Fig. 2 B).

**Figure 3:**
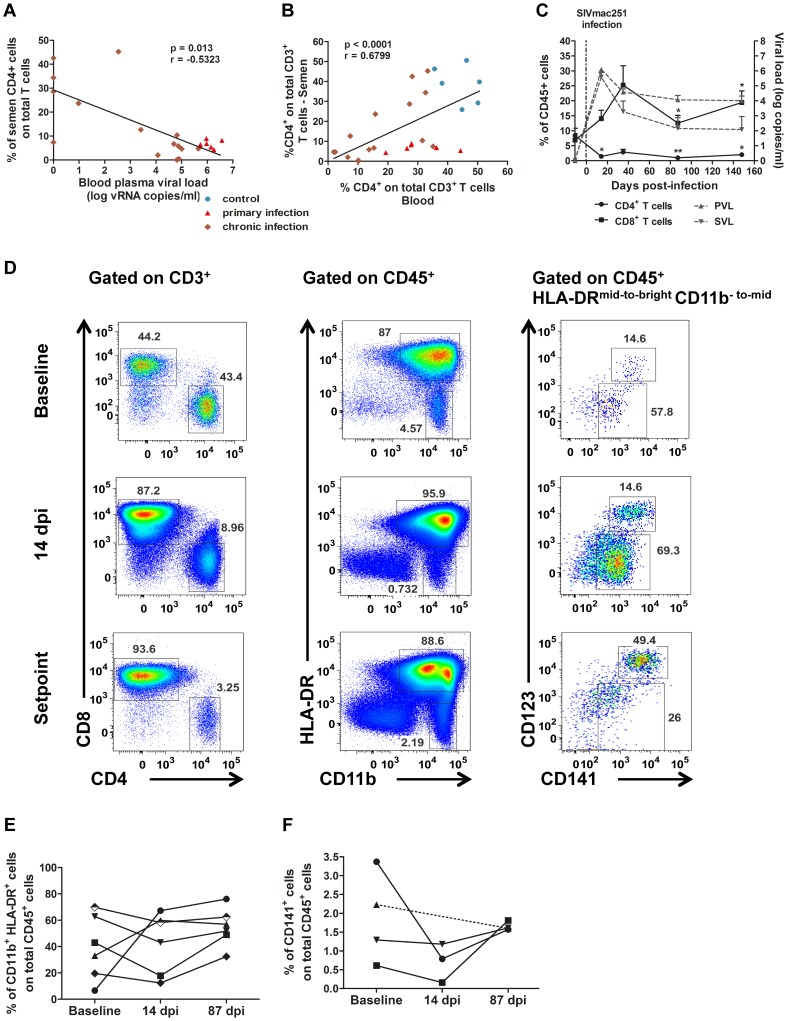
Changes in semen T cells, macrophages and DCs during SIV infection. (A) Spearman's correlation between the proportion of CD4^+^ T cells among total T cells in the semen and PVL (*n* = 6 macaques at 14 dpi – red triangle, n = 15 at chronic stage – brown diamond). (B) Spearman's correlation between the proportions of CD4^+^ T cells among total T cells for semen and blood (*n* = 6 macaques at 14 dpi – red triangle, n = 15 at chronic stage – brown diamond, and n = 6 SIV^−^ macaques). (C) RNA viral loads in blood (dotted line, grey triangle base down) and semen (dotted line, grey triangle base up) and changes in CD4^+^ (solid line, black circle) and CD8^+^ T-cell proportions (solid line, black box) during SIV infection; mean and SEM are represented. (D) Longitudinal follow-up of CD4^+^ and CD8^+^ T cells, CD11b^+^ HLA-DR^bright^ macrophages, CD11b^bright^ HLA-DR^−^ polymorphonuclear cells and CD141^+^ dendritic cells, including CD123^+^ pDCs. Dot plots from a representative animal infected with 5,000 AID_50_ IV. (E) Longitudinal follow-up of CD11b^+^ HLA-DR^bright^ macrophages in 6 macaques infected with 5,000 AID_50_ IV; each line represents an animal. (F) Longitudinal follow-up of CD123^+^ CD141^+^ pDCs in 4 SIV^+^ macaques.

In infected macaques, leukocytospermia was associated with a higher SVL than in macaques with small numbers of semen leukocytes (Mann-Whitney test, *p* = 0.0372; Fig. 2 C). In these animals, the number of CD45^+^ events was strongly correlated with SVL (Spearman correlation, *p* = 0.0008, r = 0.6858). Interestingly, 85.7% (6/7) in the primary phase of infection (10–14 dpi) displayed leukocytospermia (Mann-Whitney test, *p* = 0.0062), whereas no significant difference in the number of CD45^+^ events acquired was found between uninfected and chronically infected macaques (Fig. 2 D). The percentages of macrophages and PMN were not significantly affected by infection, but the T–cell population was significantly modified (Fig. 2 E). Semen CD4^+^ T cells were strongly and persistently depleted (2.50%±0.78%), from primary infection onwards (Mann-Whitney test, *p* = 0.0076 and 0.0077, respectively; Fig. 3 C–D). In contrast, the proportion of CD8^+^ T cells tended to be increased by the infection.

Interestingly, three of the inflammatory molecules increased by leukocytospermia were found also significantly increased in the semen of macaques during the primary infection (10–14 dpi): IP-10, RANTES and IL-8 (Mann-Whitney test, *p* = 0.01, 0.0007 and 0.0027 respectively; Fig. 2 F). Moreover, the seminal levels of RANTES and IL-8 significantly correlated to both PVL (Spearman correlation, *p* = 0.0004, r = 0.6857 and *p*<0.0001, r = 0.7445 respectively) and SVL (*p* = 0.0023, r = 0.6031 and *p*<0.0001, r = 0.7494 respectively). Their levels returned to baseline levels at chronic stages.

The percentage of CD4^+^ T cells among total semen T cells was negatively correlated with blood plasma viral load (Spearman correlation, *p* = 0.013, *r* = −0.5323; Fig. 3 A). Conversely the proportions of CD4^+^ T cells among total CD3^+^ T cells in semen and blood were positively correlated (Spearman correlation, *p*<0.0001, *r* = 0.679; Fig. 3 B).

Macrophages remained detectable in the semen at all stages of infection, with no significant change to the mean proportion among total CD45^+^ cells (Fig. 3 D–E). However, their frequency in semen was highly variable among individuals (Fig. 3 E).

Finally, CD141^+^ dendritic cells were also present in low quantity in the semen of SIV-infected macaques (Fig. 3 F). This population exhibited a tendency to decrease during primary infection (14 dpi), although non significantly, as this analysis could be performed only in 3 animals due to small proportion of these cells in semen (Wilcoxon test, *p* = 0.25, *n* = 3)

We can conclude from this first analysis that all cell types targeted by the virus are present in the semen and that significant changes in the numbers occur in macaques infected with SIV.

### Infection of semen CD4^+^ T cells and macrophages

SIVmac251 DNA, despite being detected at low frequency in total semen DNA extracted from spermatozoa and semen leukocytes could be amplified by nested PCR with primer pairs binding to *gag*. During the first month of infection, SIV DNA was detected in 87.5% of the tested animals (7 of 8 macaques), whereas 66.67% macaques at chronic stage (10 of 15 macaques at 3 months of infection or after) were tested positive. Similar results have been reported for men infected with HIV [17], [20], [21], [22], [23], [24], [25], [26], [27], [28].

In macaques, sorted CD4^+^ T cells and macrophages contained SIV-DNA (Fig. S3), as demonstrated by quantitative real-time PCR (Table 1). SIV-DNA^+^ CD4^+^ T cells and macrophages were detected as early as 7 dpi, and in 100% of the sorted fractions at 10 dpi. During chronic infection (>90 dpi), SIV-DNA remained detectable but was below the quantification limit (<90 copies). In general, infected cells were more frequently found in the CD4^+^ T cells fraction and the proportion of infected cells among the total sorted cells was higher than the macrophages fraction.

**Table 1:** Quantification of SIV DNA copies in sorted semen CD4^+^ T cells and macrophages at various stages of infection.

Animal ID	Days post-infection	Cell type	Number of sorted cells	Number of SIV DNA copies
**BB259**	7	Macrophages	16,763	<LD
	7	CD4^+^ T cells	4,892	3–90
**BB461**	7	Macrophages	25,288	<LD
	7	CD4^+^ T cells	6,305	<LD
**BB343**	7	Macrophages	2,065	3–90
	7	CD4^+^ T cells	190	3–90
**31047**	10	Macrophages	6,250	3–90
	10	CD4^+^ T cells	1,250	680.43
**10999**	10	Macrophages	13,100	3–90
	10	CD4^+^ T cells	2,286	3–90
**29860**	10	Macrophages	27,369	3–90
	10	CD4^+^ T cells	6,494	3–90
**31052**	10	Macrophages	3,759	3–90
	10	CD4^+^ T cells	906	3–90
**30602**	28	Macrophages	12,115	<LD
	28	CD4^+^ T cells	1,002	3–90
**30690**	28	Macrophages	30,836	<LD
	28	CD4^+^ T cells	8,430	3–90
**31044**	28	Macrophages	209,059	3–90
	28	CD4^+^ T cells	12,222	3–90
**30717**	135	Macrophages	4,359	<LD
	140	Macrophages	3,862	3–90
	162	Macrophages	2,853	<LD
	135	CD4^+^ T cells	689	3–90
	140	CD4^+^ T cells	298	3–90
	162	CD4^+^ T cells	759	3–90
**21362R**	135	Macrophages	623	<LD
	140	Macrophages	286	<LD
	162	Macrophages	1,037	<LD
	135	CD4^+^ T cells	73	3–90
	140	CD4^+^ T cells	27	<LD
	162	CD4^+^ T cells	102	3–90
**30838**	244	Macrophages	4,004	<LD
	249	Macrophages	4,987	<LD
	271	Macrophages	5,853	3–90
	244	CD4^+^ T cells	163	3–90
	249	CD4^+^ T cells	130	3–90
	271	CD4^+^ T cells	241	3–90
**10999**	344	Macrophages	314,146	<LD
	344	CD4^+^ T cells	10,605	3–90
**29860**	344	Macrophages	109,243	<LD
	344	CD4^+^ T cells	23,201	464.74
**30690**	367	Macrophages	35,577	3–90
	367	CD4^+^ T cells	873	3–90
**31044**	372	Macrophages	21,810	<LD
	372	CD4^+^ T cells	4,795	3–90

LD: limit of detection (3 viral DNA copies).

**Figure 4:**
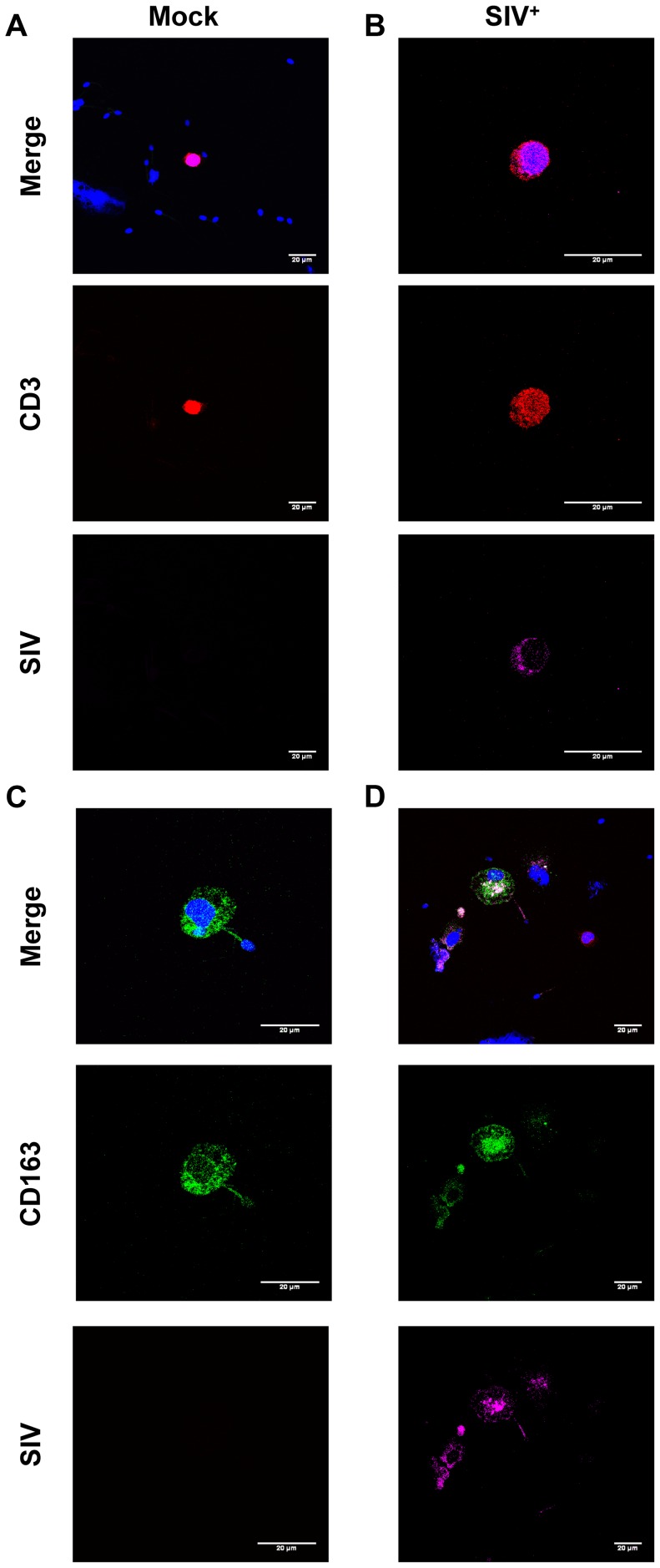
SIV antigens in semen CD4^+^ T cells and macrophages. (A–D) Immunocytofluorescence staining targeting CD3 (red), CD163 (green) and SIV Nef (pink) on cytospun CD45^+^-enriched semen cells from SIV^+^ (B,D) and uninfected macaques (A,C). Nuclei are stained using DAPI (blue), visible on merged figures (B) SIV^+^ macaque at 28 dpi. (D) SIV^+^ macaque at 65 dpi.

We also demonstrated, by immunocytofluorescence, that CD45^+^-enriched fractions of semen leukocytes contained T cells (CD3^+^) and macrophages (CD163^+^) harboring SIV proteins in macaques at 28 and 65 dpi (Fig. 4). These results are consistent with real-time PCR results for sorted cells, confirming that both CD4^+^ T cells and macrophages in semen can be infected with SIVmac251.

Finally, semen leukocytes were found to be productively infected with SIVmac251 during both primary and chronic infection. Transmission could be achieved, albeit with various degrees of efficacy, with both CD4^+^ T cells and macrophages cocultured with CEMx174 susceptible cells (Fig. S3 A–D).

### Semen CD4^+^ T-cell phenotype

As semen CD4^+^ T cells were found to be productively infected, we investigated their differentiation, activation and migratory profiles, with a view to determining their potential contribution to the mucosal transmission of HIV.

**Figure 5:**
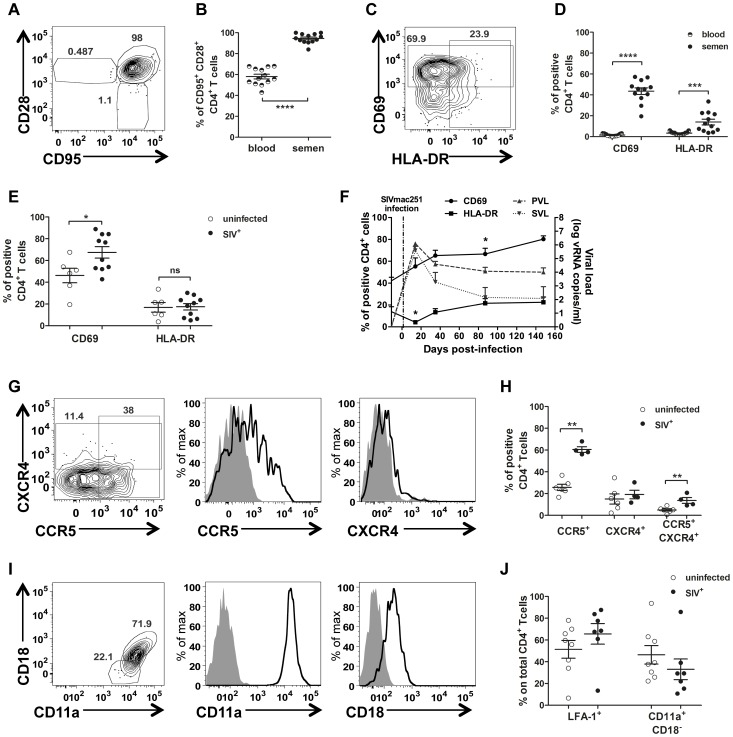
Semen CD4^+^ T-cell phenotype. (A) Gating of central memory CD4^+^ T cells (CD95^+^CD28^+)^ (B) Comparison of CD4^+^ T-cell differentiation markers between blood (half plain dot) and semen (plain dot) (*n* = 13 uninfected macaques). (C) Gating strategy for CD69 and HLA-DR expression. (D) CD69 and HLA-DR expression in CD4^+^ T cells in blood (half plain dots) and semen (plain dots) from SIV^−^ macaques (*n* = 13). (E) CD69 and HLA-DR expression in semen CD4^+^ T cells from SIV^−^ (*n* = 6, empty dots) and SIV^+^ macaques, at different stages of infection (*n* = 10, plain dots). (F) Longitudinal follow-up of CD69 (solid line, plain dot) and HLA-DR (solid line, plain box) expression in semen CD4^+^ T cells during infection (*n* = 6). Dotted lines represent PVL (plain grey triangle base down) and SVL (plain grey triangle base up) (G). CCR5 and CXCR4 expression in CD4^+^ T cells from a representative uninfected macaque. From left to right: contour plots representing outliers, overlay of CCR5 and CXCR4 staining (black line) and isotype control (solid gray curve). (H) Comparison of CCR5^+^, CXCR4^+^ and CCR5^+^ CXCR4^+^ CD4^+^ T cells between SIV^−^ (*n* = 6, open dot) and chronically SIV-infected macaques (*n* = 6, plain dot). (I) Gating strategy for LFA-1 integrin expression on CD4^+^ T cells. From left to right: contour plots representing the outliers, overlay of CD11a and CD18 staining (black line) and isotype control (solid gray curve) (J) Comparison of CD11a^+^ CD18^+^ (LFA-1) CD4^+^ T cells between SIV^−^ (*n* = 6, open dot) and chronically SIV-infected macaques (*n* = 7, plain dot). Mean and SEM are represented.

At steady state, most semen CD4^+^ T cells (94.57%±4.57%) co-expressed CD95^+^ and CD28^+^ and could therefore be considered to have a central memory phenotype (Tcm cells, Fig. 5 A), whereas such co-expression was less frequent in peripheral blood CD4^+^ T cells (58.02%±8.10, Mann-Whitney test, *p*<0.0001, Fig. 5B). Interestingly, memory CD4^+^ T cells are the preferential targets of HIV, and these cells are also the principal producers of viral particles [29], [30], [31].

CD69 expression on Tcm CD4^+^ cells is an early marker of activation and proliferation and HLA-DR is a late activation marker. In uninfected macaques, the percentage of CD69^+^ cells among semen CD4^+^ T lymphocytes was 26.8 times higher than that among blood cmCD4^+^ T cells, and the percentage of HLA-DR^+^ cells among semen CD4^+^ T cells was four times that for the corresponding lymphocytes in blood (Mann-Whitney test *p*<0.0001 and *p* = 0.0002 respectively; Fig. 5 D). Infection with SIV resulted in a significant increase in the proportion of CD69-expressing CD4^+^ T cells, beginning in primary infection (Mann-Whitney test, *p* = 0.042; Fig. 5 F). We observed a significant, transient decrease in the proportion of HLA-DR^+^ cells among semen CD4^+^ T lymphocytes at 14 dpi (Wilcoxon matched paired rank test, *p* = 0.031), followed by a rapid return to baseline levels.

We also studied the expression of CCR5 and CXCR4, two major coreceptors for HIV/SIV which are also markers of cell migration (Fig. 5 G–H). In uninfected macaques, semen T cells expressed both CCR5 and CXCR4 on their surface, with CCR5^+^ cells more numerous than CXCR4^+^ cells. A small proportion of double-positive CCR5^+^CXCR4^+^ T cells was also detected (5.512%±3.301). In infected macaques, the proportion of CCR5^+^ cells was significantly higher among CD4^+^ T cells (1.98 times higher, Mann-Whitney test, *p* = 0.0022; Fig. 5 H). This difference probably reflects the activation of T cells. Intracellular staining showed that all cells were positive for CCR5 and CXCR4, providing evidence of highly regulated surface expression (Fig. S4 A).

Finally, we studied LFA-1 expression in semen T cells. This integrin plays a crucial role in T-cell adhesion to epithelial cells and migration through mucosal tissues. Moreover, LFA-1 has been shown to be an important component of the virological synapse, which mediates the efficient cell-to-cell transmission of HIV-1 [32], [33]. LFA-1 is formed by the α-chain CD11a and the β-chain CD18. All CD4^+^ T cells express CD11a, but only a fraction of these cells express the complete form of LFA-1 (CD11a/CD18; Fig. 5 I–J). In our study, most of the CD4^+^ T cells in semen were CD11a^+^CD18^+^ (60.60%±6.18 at steady state), and the proportion of these cells was not affected by SIV infection (Fig. 5 J).

Thus, semen CD4^+^ T cells were highly activated. They expressed CCR5 and had a memory phenotype. This profile is typical of mucosal cells [34], and makes semen T cells a potential target for virus infection and replication. These lymphocytes also express migratory and adhesion factors, which might strengthen virological synapse formation and increase the capacity of the cells to migrate towards chemokine-producing tissues and, therefore, the capacity to transmit infection.

### Semen antigen-presenting cells phenotype

Macaque semen contains a population of antigen-presenting cells (APCs) that is heterogeneous in terms of morphology (using CD45 *versus* SSC-A) and HLA-DR expression (Fig. 3 and Fig. S1). We identified two major populations of APCs on the basis of the intensity of CD11b expression: a) a population of HLA-DR^bright^/CD11b^mid-to-bright^ cells, consisting mostly of macrophages, and b) a population of HLA-DR^mid-to-bright^/CD11b^-^^to mid^ cells, including dendritic cells.

**Figure 6:**
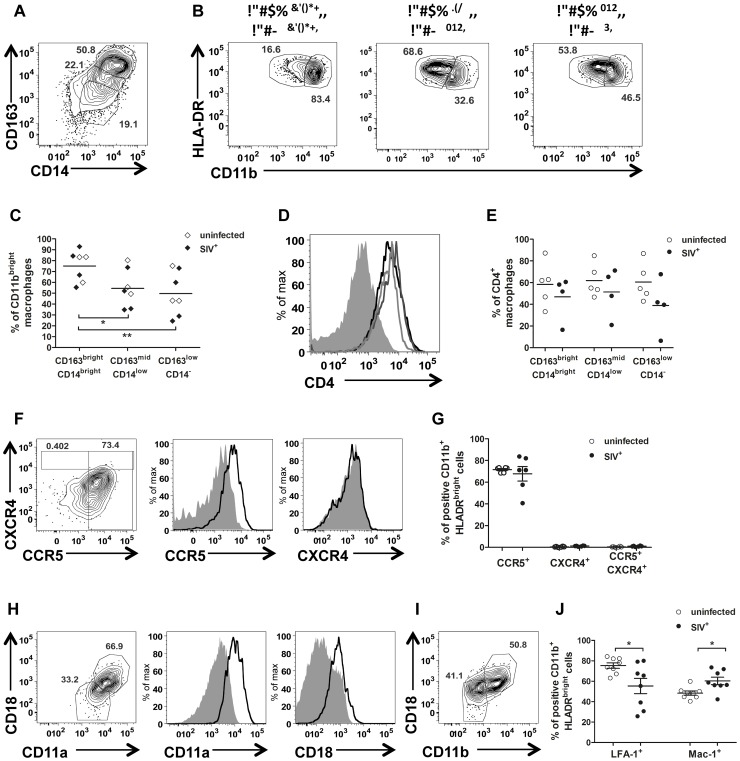
Semen macrophage phenotype. (A) Gating strategy for CD163 and CD14 expression in CD11b^+^ HLA-DR^bright^ cells. (B) Intensity of CD11b expression by subset (CD163^bright^CD14^bright^, CD163^bright^CD14^low^ and CD163^low^CD14^−^ respectively), in a representative animal. (C) Proportion of CD11b^bright^ cells in each subset in 5 SIV^−^ (open dot) and 4 SIV^+^ animals (plain dot). (D) CD4 expression in each subset from a representative uninfected animal (CD163^bright^CD14^bright^: black line, CD163^bright^CD14^low^: dark grey line and CD163^low^CD14^−^: light grey line). The solid grey curve represents the negative control (no anti-CD4 staining). (E) Comparison of CD4 expression in each subset between 4 SIV^−^ (open dot) and 6 SIV^+^ macaques (plain dot). (F) CCR5 and CXCR4 expression in a representative SIV^−^ macaque. From left to right: contour plots representing the outliers, overlay of CCR5 and CXCR4 staining (black line) and isotype control (solid gray curve). (G) Comparison of CCR5^+^, CXCR4^+^ and CCR5^+^ CXCR4^+^ macrophages between SIV^−^ (*n* = 6, open dot) and SIV^+^ macaques (*n* = 6, plain dot). (H) Gating strategy for LFA-1 integrin expression. From left to right: contour plots representing outliers, overlay of CCR5 and CXCR4 staining (black line) and isotype control (plain gray curve). (I) Gating strategy for Mac-1 integrin expression. (J) Comparison of LFA-1 and Mac-1 expression by macrophages between 6 SIV^−^ (open dot) and 8 SIV^+^ (plain dot) macaques. SIV^+^ macaques were all at chronic stage of infection. Mean and SEM are represented.

Macrophages were the most abundant HIV/SIV target cell in semen. Variable intensities of CD163, CD14 and CD11b expression were observed (Fig. 6 A), resulting in the definition of three different subsets. The most frequent of these subsets was CD163^bright^CD14^bright^ cells (31.59%±3.51 of CD11b^+^ HLA-DR^+^ cells), 74.27%±5.01 of which were CD11b^bright^ (Fig. 6 B–C). This profile is typical of activated macrophages. The second subset was CD163^mid^CD14^low^ cells (27.24%±2.39), and the third was CD163^low^CD14^−^ cells (mean 29.26%±3.84). This last subset, which may also include dendritic cells, contained equal proportions of CD11b^bright^ and CD11b^mid^ cells (57.44%±6.80 and 53.11%±7.06, respectively). The proportion of CD11b^bright^ cells in subset 1 was significantly different from those in subsets 2 and 3 (Mann-Whitney test, *p* = 0.011 and *p* = 0.003, respectively). All semen macrophages expressed CD4 (Fig. 6 D–E). We also found no significant difference in macrophages CD4 expression between uninfected and SIV-infected macaques (Fig. 6 E).

Interestingly, HLA-DR expression was found to be decreased at 14 dpi, both on semen macrophages and on CD4^+^ T cells (Fig. S5 A, Fig. 3 D). By contrast, CD11b had increased (83.70%±2.03% of CD11b^bright^; Wilcoxon test, *p* = 0.0313, Fig. S5 B). The concentrations of the pro-inflammatory chemokines MCP-1, MIP-1β and IL-8, which are produced principally by macrophages, also decreased transiently at this time point (Fig. S5 C). These observations indicate that the acute peak of SVL is accompanied by major changes to the activation and maturation profiles of semen macrophages.

Semen macrophages strongly expressed CCR5 at the membrane, and this expression was not affected by SIV infection (Fig. 6 F–G). By contrast, the proportion of CXCR4^+^ cells was significantly higher in infected than in uninfected macaques (Mann-Whitney test, *p* = 0.011), although this proportion remained low (2.96%±0.31). As for CD4^+^ T cells, almost 100% of semen macrophages contained intracellular CCR5 and CXCR4, indicating high levels of turnover for these molecules (Fig. S5 B).

Most semen macrophages expressed LFA-1 (76.93%±2.79 at steady state). The expression of this molecule was significantly decreased by SIV infection (Mann-Whitney test, *p* = 0.049; Fig. 6 H, J). CD11b^+^ macrophages also expressed Mac-1 (49.08%±2.678, Fig. 6 I). This integrin is formed by the α-chain CD11b and the β-chain CD18. The mean proportion of double-positive cells was increased significantly (*p* = 0.028) by SIV infection (Fig. 6 J).

## Discussion

We report here in a macaque model of transmission the first comprehensive characterization of semen leukocytes with the potential to transmit HIV/SIV infection across mucosal barriers. This study was carried out with cynomolgus macaques infected with pathogenic SIVmac251, which is recognized as the most relevant experimental model for studies of HIV infection pathogenesis and mucosal transmission [13], [35], [36], [37]. However, the fact that we used a unique virus isolate, does not allow full representation of the diversity of the HIV-1 populations found in the semen of infected men. Although it is dual-tropic, it is well established that SIVmac251 shows a high tropism for macrophages [38] which is not the case for a significant proportion of founder HIV. Therefore we cannot exclude that our model over estimates the role of macrophage-tropic strains of HIV-1 in transmission.

Macaque semen was found to be very similar to human semen, with similar distributions and phenotypes of semen leukocytes in these two species. Moreover, the dynamics of viral RNA shedding in the semen of SIVmac251-infected macaques was similar to that reported for men. Seminal plasma viral load was correlated with blood plasma vRNA, although some discrepancies were observed in untreated animals.

Leukocytospermia is frequent in humans and macaques and has been reported to be associated with higher levels of HIV DNA in humans, suggesting that semen leukocytes may contribute to virus transmission across mucosal surfaces [3]. We show here that the presence of large numbers of leukocytes in semen is associated with high levels of inflammation markers (IL-6, IL-8, MIP-1β, MCP-1, RANTES and IP-10). Concentrations of these cytokines were correlated with blood and/or seminal plasma viral load. Interestingly, in SIV-infected macaques, leukocytospermic animals had higher seminal vRNA. Moreover, during primary SIV infection, the semen is highly inflammatory and contains large numbers of macrophages, a major target of HIV.

As in other compartments, CD4^+^ T cells were rapidly and profoundly depleted in the semen of SIV-infected macaques. These cells have a central memory phenotype (CD95^+^CD28^+^) and express CCR5, a profile typical of resident mucosal T cells [31], [39], [40]. A large proportion of cells expressed CD69, an activation marker observed at all stages of infection. If infected, semen CD4^+^ T cells should be able to produce replicative viral particles.

Despite the high degree of CD4^+^ T-cell depletion in semen, SIV DNA was detected in these cells at all stages of infection. Only a few CD4^+^ T cells could be sorted from semen, but these cells transmitted the infection when cocultured *in vitro* with a permissive cell line, demonstrating their considerable capacity to produce infectious SIV. However, in the context of chronic infection, CD4^+^ T cells are present in very small numbers and therefore probably have a very small impact on virus transmission. Further studies are required to confirm this hypothesis.

In our study, semen macrophages formed a heterogeneous population, with different levels of CD163, CD14 and CD11b expression. This population also expressed markers of activation and migration. Interestingly, semen HLA-DR^+^ APCs contain SIV DNA at all stages of infection, including the first weeks after infection. Moreover, like T cells, these cells were able to transmit infection to CEMx174 cells when collected at the primary and chronic stages of infection. Thus, semen APCs are also productively infected and produce replication-competent viral particles. Macrophages from chronically infected and leukocytospermic individuals are therefore major candidates for involvement in cell-associated virus transmission.

We also identified other types of APC in semen. This is the first study to report the presence of dendritic cells (DCs), including pDCs, in the semen of infected macaques. More studies are required to characterize semen DCs, particularly for the CD141^+^ CD123^−^ subpopulation, and their role in the dissemination of viral particles requires investigation.

We found that a large proportion of semen CD4^+^ T cells and macrophages expressed CCR5, one of the co-receptors of HIV/SIV. The expression of this coreceptor was significantly stronger in infected than in uninfected macaques. This predominance of CCR5^+^ viral target cells might also account for most of the transmitted viral founders, after mucosal exposure, being R5 strains. It also provides support for the role of cell-associated virus transmission, because semen leukocytes have a migratory and mucosal profile, and are therefore able to migrate to tissues producing RANTES, MIP-1α and MIP-1β, such as the cervico-vaginal mucosa [41]. Furthermore, semen T cells and macrophages express high levels of LFA-1 and/or Mac-1 integrins. Both play an important role in leukocyte adhesion to epithelial cells and transmigration. LFA-1 has also been described as a key player in virological synapse formation and virus transmission [32], [33].

Taken together, these data suggest that infected semen leukocytes may play a role in mucosal transmission, even if present in very small numbers. This hypothesis is supported by the observation that infection may be initiated by a very small number of transmission events [42], [43], [44], [45]. Mucus, low pH and natural microbicidal molecules secreted by the genital and rectal mucosa may be less effective against infected cells than against free viral particles. In this scenario, semen leukocytes may act as Trojan horses, protecting cell-associated virus from host mucosal defenses [3].

It remains nevertheless hard to evaluate the relative impact of cell-associated HIV in mucosal transmission in comparison to free viral particles. Although viral DNA is detected in semen leukocytes at all stages of infection, in our model, SIV-DNA is present in quantifiable and substantial amounts only during the acute phase of infection. The first weeks of infection may therefore represent a restricted window of opportunity for cell-associated virus transmission, whereas infected semen leukocytes may not play a substantial role during the chronic stage.

## Materials and Methods

### Ethics statement

Adult cynomolgus macaques (*Macaca fascicularis*) were imported from Mauritius and housed in the facilities of the “Commissariat à l'Energie Atomique et aux Energies Alternatives” (CEA, Fontenay-aux-Roses, France). Non-human primates (NHP, which includes *M. fascicularis*) are used at the CEA in accordance with French national regulation and under national veterinary inspectors (CEA Permit Number A 92-032-02). The CEA is in compliance with Standards for Human Care and Use of Laboratory of the Office for Laboratory Animal Welfare (OLAW, USA) under OLAW Assurance number #A5826-01. All experimental procedures were also conducted accordingly to European guidelines for animal care (European directive 86/609, “Journal Officiel des Communautés Européennes”, L358, December 18, 1986). The use of NHP at CEA is also in accordance with recommendation with the newly published European Directive (2010/63, recommendation N°9). No suffering was specifically associated with vaginal treatment of macaques. The animals were used under the supervision of the veterinarians in charge of the animal facility. This study was part of the European microbicides project Combined Highly Active Anti-Retroviral Microbicides (CHAARM), which NHP studies were accredited by ethical committee “Comité Régional d'Ethique pour l'Expérimentation Animale Ile-De-France Sud” under statement numbers 12-048 (December 6^th^ 2012) and 12-103 (December 31^st^ 2012).

### Animals, infection and sample collection

Adult male macaques were infected by intravenous or intrarectal inoculation with a single dose of 50–5,000 50% animal infectious doses (AID_50_) of SIVmac251 [46]. Semen and blood were collected from animals sedated by a 5 mg/kg intramuscular injection of chlorhydrate Tiletamine (50 mg) combined with chlorhydrate Zolazepan (50 mg) (Virbac, Carros, France).

Ejaculation was performed by intrarectal electrostimulation of nervous centers near the prostate, with a probe (12.7 mm diameter) lubrified with conductor gel, and an AC-1 electroejaculator (Beltron Instrument, Longmont, USA). Sequential stimulations were performed, with a pattern of 6 cycles, each cycle consisting of nine two-second stimulations followed by a tenth stimulation lasting 10 seconds. The voltage was increased every two cycles (1–3 V for the first two cycles, 2–4 V volts for the third and fourth cycles and 6–8 V for the last two cycles). If a complete ejaculate had not been obtained after six cycles of stimulation, a 7^th^ cycle of stimulation at 7–10 V was performed. The complete ejaculate was immediately diluted in 1.2 ml of phosphate-buffered saline (PBS) and centrifuged.

Blood sample were collected into K_3_EDTA tubes (BD Biosciences, Le Pont de Claix, France).

### Seminal plasma and cell preparation

Seminal plasma was isolated from total semen immediately after collection, by centrifugation for 15 minutes at 775× *g*. The seminal cell pellet was resuspended in 14 ml of complete medium, consisting of RPMI-1640 medium enriched in glutamine (Invitrogen, Carlsbad, USA) supplemented with a mixture of penicillin, streptomycin and neomycin (Invitrogen) and 10% FCS (Lonza, Allendale, USA), and kept at room temperature for no more than one hour. Cells were then centrifuged for 10 min at 1,500× *g*, filtered through a sieve with 70 µM pores and washed with 5 ml of PBS supplemented with 10% FCS.

### Blood and semen RNA viral load quantification

Blood plasma was isolated from EDTA-treated blood samples by centrifugation for 10 min at 1,500× *g*, and stored frozen at −80°C. Seminal plasma samples were maintained on ice for no more than one hour and were frozen at −80°C. Semen vRNA was prepared from 500 µl of seminal plasma with commercial kit (QIAamp UltraSens, Qiagen, Courtaboeuf, France), according to the manufacturer's instructions. RT-PCR on blood and seminal plasma RNA was performed as previously described (Karlsson, 2007, J Virol). The quantification limit (QL) was estimated at 111 copies/ml and the detection limit (DL) was estimated at 37 copies/ml. Samples from chronically infected macaques on antiretroviral treatment (ART) were treated in the same way, increasing the amount of plasma to increase sensitivity (QL and DL of 37 and 12.3 copies of vRNA/ml, respectively).

### Phenotypic characterization of semen leukocytes

Staining was performed on either whole blood or PBMC. Except for whole-blood assays, staining was performed after the saturation of Fc receptors by incubation with healthy macaque serum (produced in-house) for 1 h at 4°C. Amine-reactive blue dye (Live/dead Fixable, Life Technologies) was used to assess cell viability and to exclude dead cell from the analysis. Cells were stained with monoclonal antibodies by incubation for 30 min at 4°C, washed in PBS/10% FCS and fixed in commercial fixation solution (CellFIX, BD Biosciences). Five different antibody panels were used (see Protocol S1). Corresponding isotype controls for CD163, CD14, CCR5, CXCR4, CD11a and CD18 were used at the same concentrations as the reference antibody. Acquisition was performed on a BD LSRII machine equipped with four lasers (355, 405, 488 and 633 nm), with Flowjo v9 (Tree Star, Ashland, OR) used for analysis.

Semen sample were considered leukocytospermic if it was possible to acquire a minimum of 50,000 CD45^+^ events per ml on flow cytometry (consisting of 10,000 events per ejaculate, Fig. S6). This cut-off was defined as the minimal required number of acquired CD45^+^ events to perform statistically relevant analysis of the different populations of leukocytes.

### Sorting of semen CD4^+^ T cells and antigen-presenting cells

Total semen cells were filtered and washed (see above), and then incubated for 15 min at 4°C with 20 µl of anti-CD45 magnetic beads (Miltenyi Biotec) and washed once with 2 ml of cold PBS supplemented with 0.5% BSA and 2 mM EDTA (sorting buffer). The CD45^+^ cell fraction was then enriched by magnetic bead sorting, with LS columns (Miltenyi Biotec), used according to the manufacturer's instructions. Cells were eluted in 4 ml of sorting buffer. Cells were stained as described above, using amine-reactive blue dye (Life Technologies) to identify the dead cells, and the same antibodies as for leukocyte phenotyping: CD45, CD3, CD8, CD4 and HLA-DR. Cells were washed twice and stored at 4°C in PBS/10% FCS. Following the magnetic bead-based enrichment process, CD4^+^ T cells and HLA-DR^+^ APCs were sorted by simultaneous four-way sorting on a FACSAria flow cytometer (BD Biosciences). The enrichment of cell fractions was estimated by flow cytometry with BDDiva (BD Biosciences) and FlowJo software (see Fig. S2).

### Detection of SIV DNA in the cellular components

After semen collection and the separation of the cellular components from the seminal plasma, the cells were centrifuged and the cell pellet was kept at −80°C until further tests. Nested PCR targeting SIVmac251 *gag* was performed as previously described [47]. Twenty tests were carried out for each semen DNA sample. A second amplification was then carried out, as we previously described [14].

After the sorting of CD4^+^ T cells and macrophages, the cells were washed once in PBS and centrifuged for 10 min at 1,500× *g*. The supernatant was discarded and the dry cell pellets were frozen at −80°C. PCR was directly performed on cell lysates as we previously described [37]. The number of cells was determined by amplifying the CCR5 gene (see Protocol S2) with serial 10-fold dilutions of SIV-negative PBMCs (starting with 1 million cells) as a standard and a lysate of SIV-negative PBMCs and nuclease-free water as the negative control. SIV DNA was quantified with serial dilutions over five orders of magnitude of a pCR4-TOPO plasmid (Invitrogen) containing the SIVmac251 *gag* cDNA sequence and diluted in SIV-negative PBMCs as the standard. The quantification threshold was 90 SIV-DNA copies and the detection threshold varied between 1 and 3 copies of SIV-DNA (see Figure S7).

### Coculture of sorted semen leukocytes with CEMx174

After sorting, semen CD4^+^ T cells and macrophages were washed once with 10% FCS in PBS and transferred to a U-bottomed 96-well plate. CEMx174 cells were added to each well, the number of cells added being three times the number of sorted cells. If this number was below 10,000 cells, we added a minimum of 50,000 CEMx174 cells per well. Cells were cultured in a final volume of 250 µl of complete medium (see above), at 37°C, under an atmosphere containing 5% CO_2_. The positive control was CEMx174 cells cultured with 50 µl of highly concentrated SIVmac251 particles, and the negative control was CEMx174 cells alone. Cells were passaged three or four days after the initiation of the coculture and then every two or three days thereafter. Coculture was stopped after 8 days. At each passage, half the cell suspension was replaced with fresh medium. Supernatants and cell pellets were cryopreserved at −80°C until further analysis. SIV-DNA and SIV-RNA were quantified as described above.

### Immunofluorescence staining of cytospun semen cells

Cells were washed once in PBS, then diluted in a maximal volume of 450 µl and cytospun on Superfrost slides at 500T for 10 min, in a Cytospin 4 Cytocentrifuge (Thermo Shandon, Thermo Fisher Scientific, Waltham, USA), at a concentration of 200,000 cells per spot. Cytospun cells were allowed to dry at room temperature for two hours and were then fixed by incubation in a mixture of 50% ice-cold methanol and 50% ice-cold acetone for 15 min. Slides were then allowed to dry at room temperature for 20 min and frozen at −20°C for later use.

Before staining, the slides were thawed and allowed to dry at room temperature for 20 min. They were then washed three times, for 5 min each, in PBS. Cells were permeabilized by incubation with 0.025% Triton in PBS for 5 min and the slides were then washed three times, for five minutes each, in PBS. Non specific binding sites were saturated by incubation for 1 hour at room temperature with 5% BSA and 10% macaque serum in PBS. Immunofluorescence staining was performed with the following antibodies: anti-CD3 Alexa Fluor 700 (BD Biosciences, clone SP34-2), anti-CD163 Alexa Fluor 488 (BD Biosciences, clone GHI/61) and anti-SIV nef (NIBSC, Center for AIDS Reagents, clone KK75) and anti-SIV gp41 (NIBSC, Center for AIDS Reagents, clone KK7a) both coupled with Alexa Fluor 594, in 10% BSA/0.05% Tween 20 in PBS, for 30 min, at room temperature, in a dark chamber. Slides were washed three times with 0.05% Tween 20 in PBS and three times in PBS alone. Finally, slides were mounted in anti-fade mounting medium which stained nuclei using DAPI (ProLong, Invitrogen) and stored at 4°C until further analysis.

Images were acquired with a Leica confocal microscope (equipped with 4 lasers) and analyzed with ImageJ software. The final images were generated with Adobe Photoshop.

### Cytokine quantification in semen

Concentrations of IL-6, IL-8, MIP-1β and MCP-1 in seminal plasma were determined with the Milliplex Map Non-Human Primate Cytokine Magnetic Bead Panel - Premixed 23-plex (Merck Millipore, Darmstadt, Germany). The concentrations of RANTES and IP-10 were determined with a 2-plex Milliplex kit. Assays were performed in duplicate, with 25 µl of seminal fluid. Samples were thawed at room temperature and centrifuged for 10 min at 1,500× *g* to harvest any cellular components. Immunoassays were performed according to the manufacturer's instructions. Data were acquired with a Bio-Plex Instrument 200 and analyzed with Bio-Plex Manager Software version 6.1 (Bio-Rad, Hercules, USA).

### Data visualization and statistical analysis

All data visualization and statistical analyses were carried out with GraphPad Prism 5.03 software (GraphPad software, La Jolla, USA). Nonparametric Spearman's rank correlation tests were used to investigate the relationships between parameters. The nonparametric Mann-Whitney test was used to compare groups of macaques, and the nonparametric Wilcoxon rank sum test was used to compare data from same macaques at different time points, before and after SIV infection. *P* values of 0.05 or less in two-tailed tests were considered significant, *:*p*<0.05, **: *p*<0.01, ***: *p*<0.001, ****: *p*<0.0001.

## Supporting Information

Figure S1 **Gating strategy for semen leukocyte characterization.** (A) Exclusion of all events other than those for leukocytes; from left to right, doublets, cell debris and dead cells are excluded and leukocytes are identified with the pan-leukocyte marker CD45. (B) The SSC-A *versus* CD45 gate distinguishes lymphocytes from macrophages and polymorphonuclear cells on the basis of morphology. (C) CD11b and HLA-DR distinguish HLA-DR^bright^ CD11b^mid-to-bright^ antigen-presenting cells from CD11b^bright^ HLA-DR^negative-to-low^ polymorphonuclear cells. (D) CD3^+^ T cells are gated against HLA-DR, and CD4^+^ T cells are separated from CD8^+^ T cells. (E) Gating strategy for dendritic cell identification: after gating on CD45 and SSC-A, all cells positive for theCD3, CD8, CD20 lineage are excluded and HLA-DR^mid–to-bright^ CD11b^low-to-negative^ cells are gated. Cells negative for CD14 and CD163 are selected, most being CD141^+^ (BDCA3) dendritic cells, although some are CD123^+^ pDCs.
(TIF)

Figure S2 **Purity of the sorted CD4^+^ T-cell and macrophage fractions.** (A) Control of the purity of the sorted CD4^+^ T-cell fraction. (B) Control of the purity of the sorted HLA-DR^+^ macrophage fraction.
(TIF)

Figure S3 **Macroscopic evidence for virus transmission and quantification of SIV DNA copies in cocultures of sorted semen CD4^+^ T cells and macrophages.** (A) Negative (culture medium only) and positive (SIVmac251) controls at 8 days of coculture. (B) Coculture of CEMx174 cells with semen macrophages from 1 macaque at 10 dpi (#31047) and 2 macaques with chronic infection (#21362R and 30717). (C) Coculture with semen CD4^+^ T cells from 1 macaque at 10 dpi (#31047) and 1 macaque at 35 dpi (#31044). (D) Number of SIV DNA copies in cells from cocultures of sorted semen CD4^+^ T cells and macrophages with CEMx174 cells (log copy number per one million cells). Each dot and line represents one set of conditions.
(TIF)

Figure S4 **Intracellular CCR5 and CXCR4 in semen CD4^+^ T cells and macrophages.** (A) Gating strategy for CD4^+^ T cells. (B) Gating strategy for macrophages. Gating strategy based on the isotype controls staining (the background of the PE-isotype is different from the extracellular staining).
(TIF)

Figure S5 **Changes to semen macrophages during SIV infection.** (A) Longitudinal follow-up of the mean fluorescence intensity (MFI) for HLA-DR in infected macaques (at 14 and 35 dpi, data were available for only 4 macaques). (B) Longitudinal follow-up of the proportion of CD11b^bright^ ^and^ ^mid^ among total CD11b^+^ HLA-DR^bright^ cells (C) Dynamics of seminal plasma MCP-1, Il-8 and MIP-1b concentrations, as determined with Luminex technology. (A–C) Dotted lines represent PVL (plain triangle base dow) and SVL (plain triangle base up).
(TIF)

Figure S6 **Definition of leukocytospermia.** Number of total acquired CD45^+^ events in non-leukocytospermic and leukocytospermic macaques (n = 15 animals in each group). Mean and SEM are represented. A cut-off is defined at 10,000 positive events.
(TIF)

Figure S7 **Quantification of SIV DNA in semen leukocytes by real-time PCR.** Quantification of SIV DNA copies using a standard dilution of a SIV *Gag* plasmid diluted in macaques genomic DNA. The data of four different experiments conducted in duplicates are represented. Linear regression is represented with mean and SEM (95% Cl.). %CV: coefficient of variation as a percentage (%CV = 100*standard deviation/mean). The limit of quantification (LQ) is 90 SIV DNA copies (%CV = 1.57). The limit of detection (LD) is 3 SIV DNA copies. Background threshold is defined at 40 threshold cycle (C_T_, Y axis).
(TIF)

Protocol S1 **Antibodies panel for semen T cells and antigen-presenting cells phenotyping.** No legend.
(DOCX)

Protocol S2 **Primers and Taqman probe specific to cynomolgus macaque CCR5 gene.** Nucleotide sequence.
(DOCX)

Table S1 **Influence of SIV infection on molecules more abundant in leukocytospermic semen.** * **:** Mann-Whitney test *p* control *versus* chronic infection. Mean and SEM are specified. Normal semen group: n = 12, leukocytospermic semen group: n = 13.
(DOCX)
