# Supplementary figures and images for: Semen CD4+ T Cells and Macrophages Are Productively Infected at All Stages of SIV infection in Macaques
Source: PLoS Pathog. 2013 Dec 12;9(12):e1003810. doi: 10.1371/journal.ppat.1003810 (PMC3861532; doi:10.1371/journal.ppat.1003810)

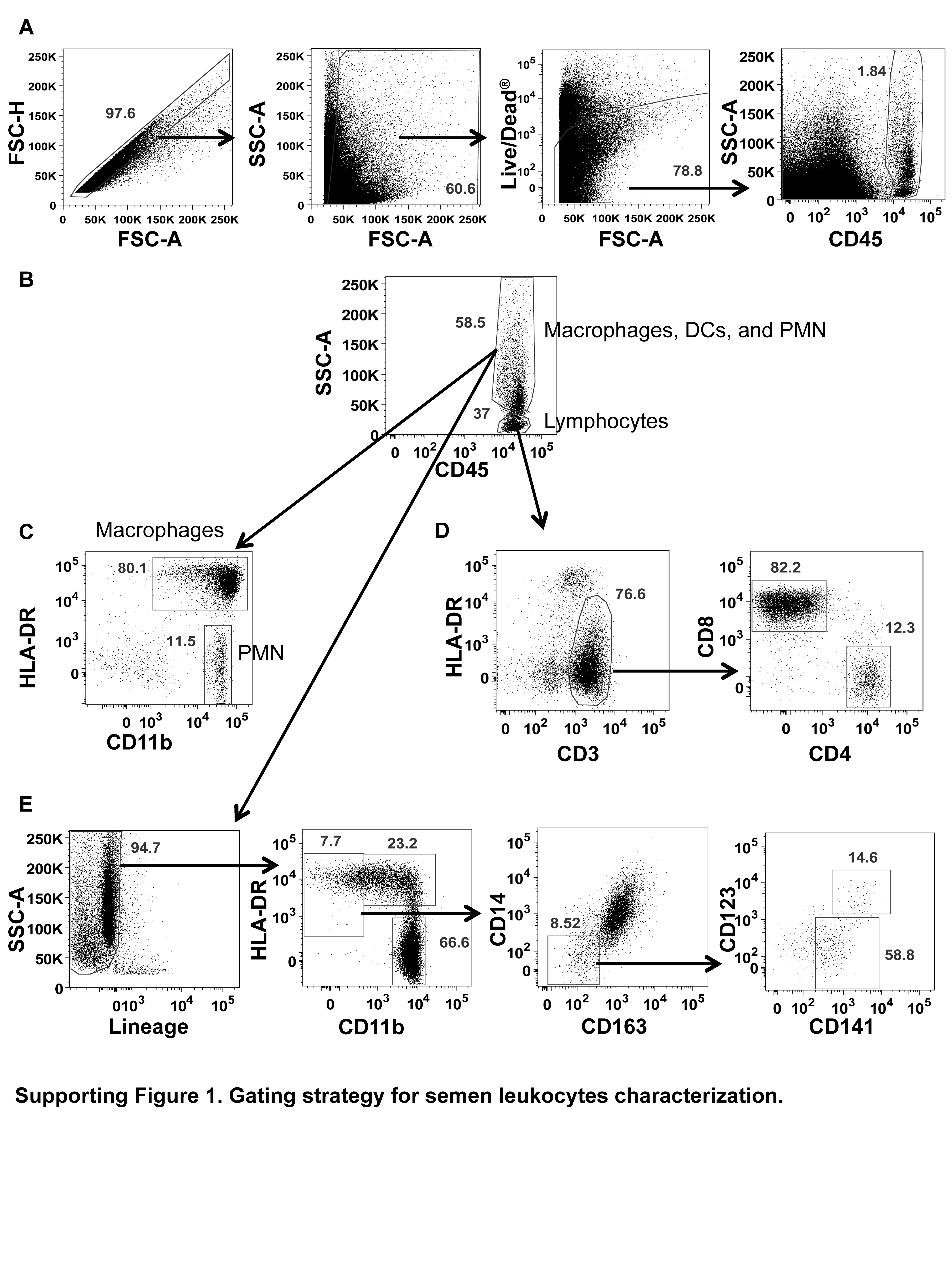

Supplement: Figure S1 — Gating strategy for semen leukocyte characterization. (A) Exclusion of all events other than those for leukocytes; from left to right, doublets, cell debris and dead cells are excluded and leukocytes are identified with the pan-leukocyte marker CD45. (B) The SSC-A versus CD45 gate distinguishes lymphocytes from macrophages and polymorphonuclear cells on the basis of morphology. (C) CD11b and HLA-DR distinguish HLA-DRbright CD11bmid-to-bright antigen-presenting cells from CD11bbright HLA-DRnegative-to-low polymorphonuclear cells. (D) CD3+ T cells are gated against HLA-DR, and CD4+ T cells are separated from CD8+ T cells. (E) Gating strategy for dendritic cell identification: after gating on CD45 and SSC-A, all cells positive for theCD3, CD8, CD20 lineage are excluded and HLA-DRmid–to-bright CD11blow-to-negative cells are gated. Cells negative for CD14 and CD163 are selected, most being CD141+ (BDCA3) dendritic cells, although some are CD123+ pDCs. (TIF) [file ppat.1003810.s001.tif]

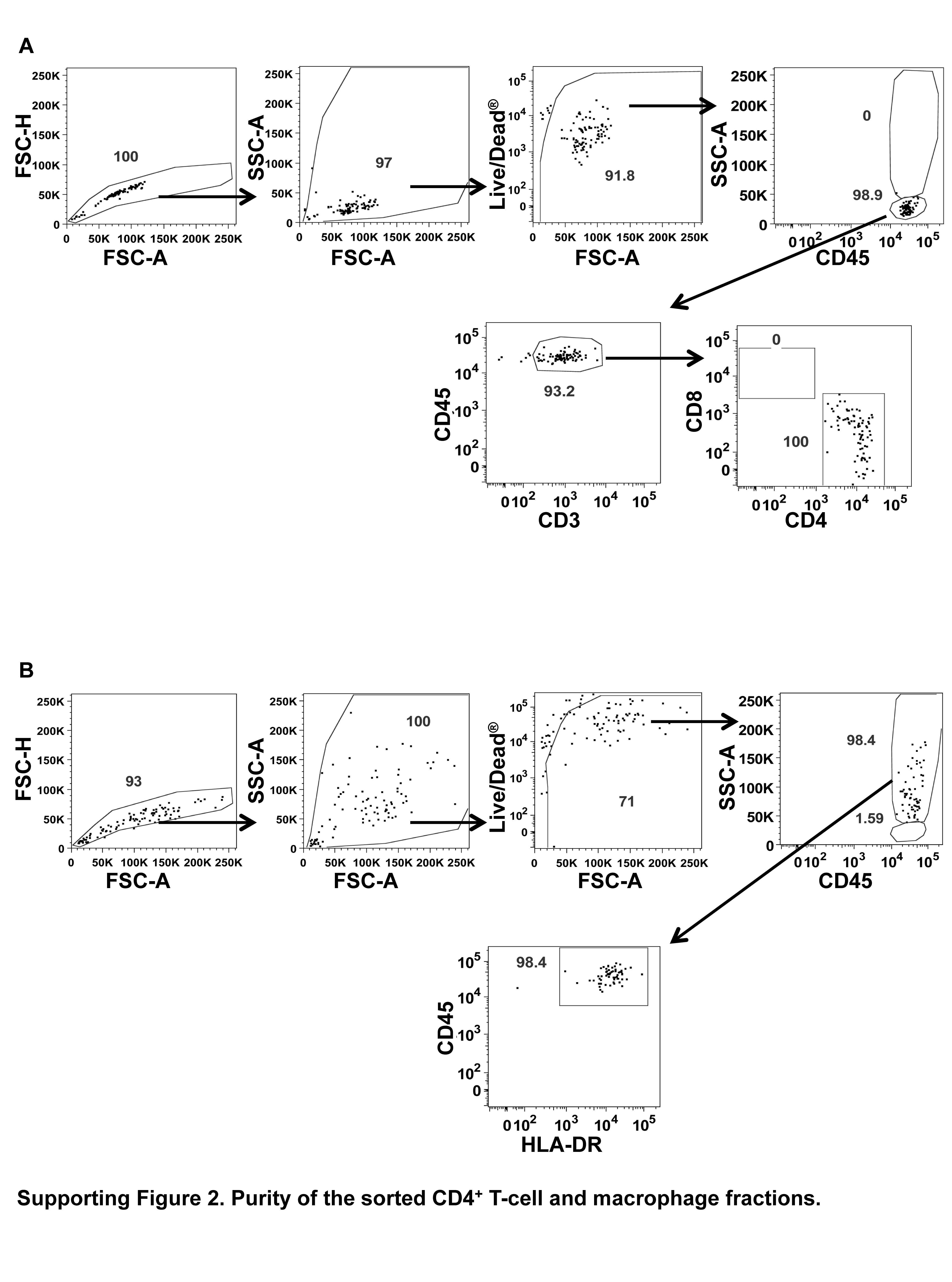

Supplement: Figure S2 — Purity of the sorted CD4+ T-cell and macrophage fractions. (A) Control of the purity of the sorted CD4+ T-cell fraction. (B) Control of the purity of the sorted HLA-DR+ macrophage fraction. (TIF) [file ppat.1003810.s002.tif]

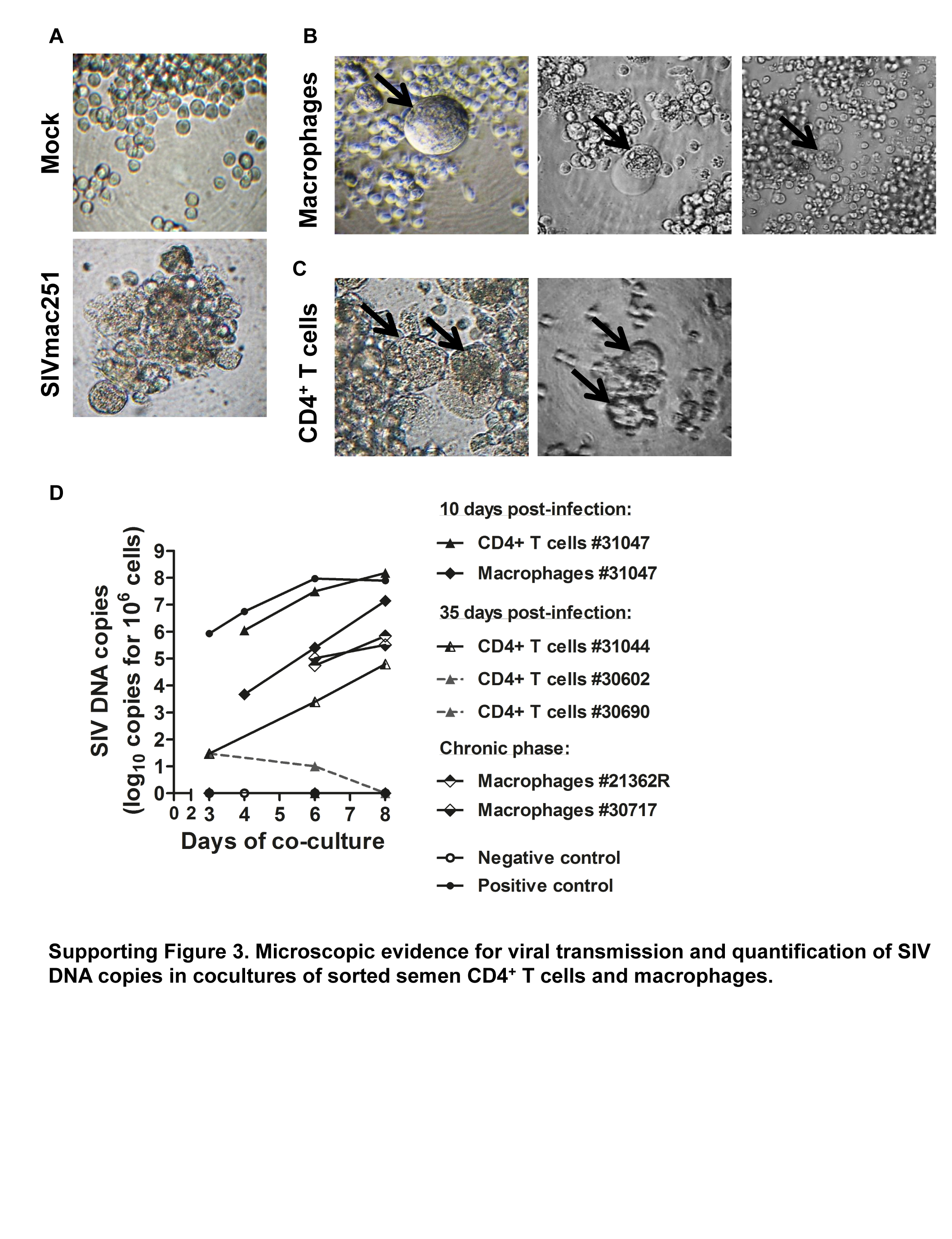

Supplement: Figure S3 — Macroscopic evidence for virus transmission and quantification of SIV DNA copies in cocultures of sorted semen CD4+ T cells and macrophages. (A) Negative (culture medium only) and positive (SIVmac251) controls at 8 days of coculture. (B) Coculture of CEMx174 cells with semen macrophages from 1 macaque at 10 dpi (#31047) and 2 macaques with chronic infection (#21362R and 30717). (C) Coculture with semen CD4+ T cells from 1 macaque at 10 dpi (#31047) and 1 macaque at 35 dpi (#31044). (D) Number of SIV DNA copies in cells from cocultures of sorted semen CD4+ T cells and macrophages with CEMx174 cells (log copy number per one million cells). Each dot and line represents one set of conditions. (TIF) [file ppat.1003810.s003.tif]

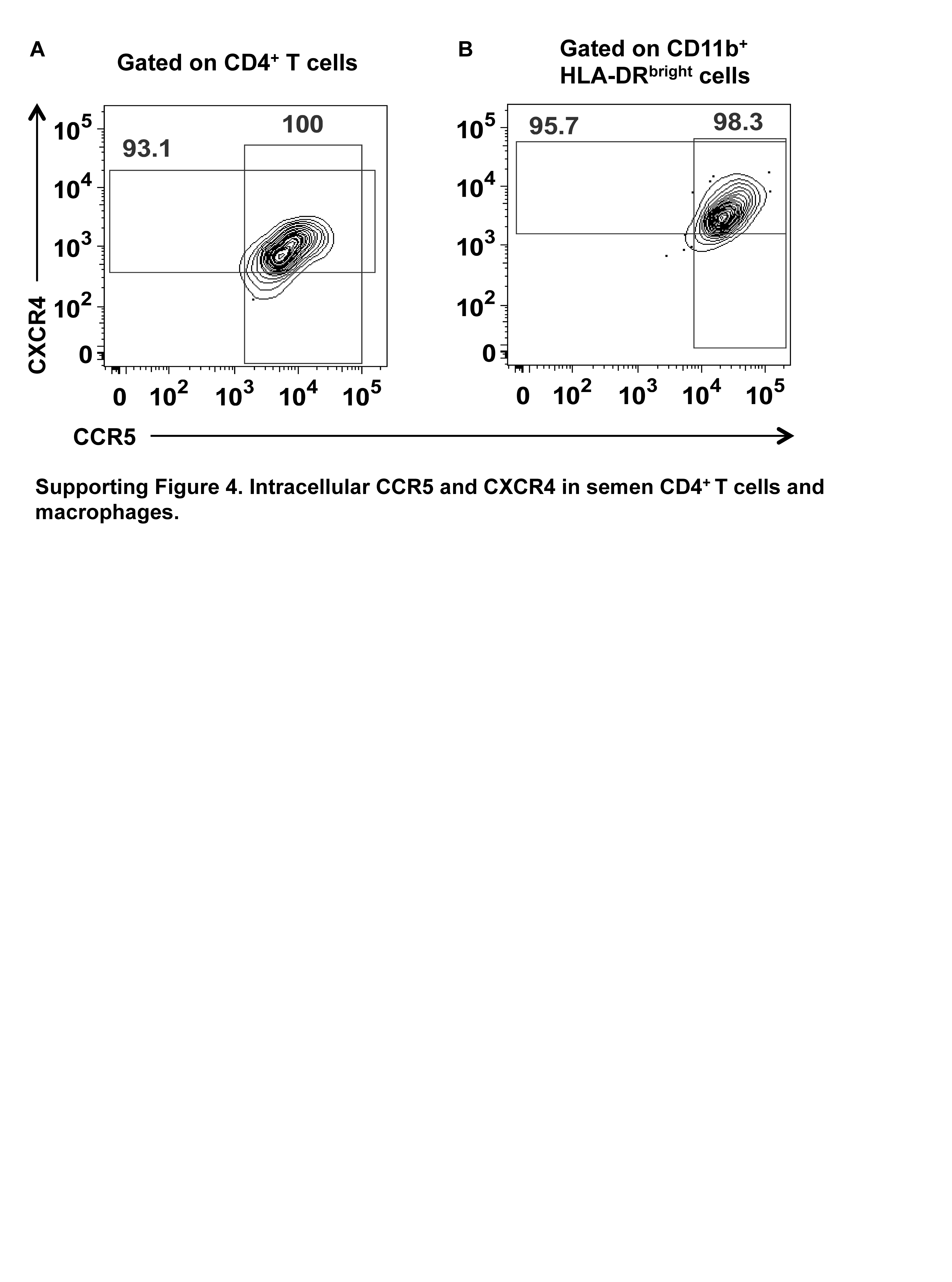

Supplement: Figure S4 — Intracellular CCR5 and CXCR4 in semen CD4+ T cells and macrophages. (A) Gating strategy for CD4+ T cells. (B) Gating strategy for macrophages. Gating strategy based on the isotype controls staining (the background of the PE-isotype is different from the extracellular staining). (TIF) [file ppat.1003810.s004.tif]

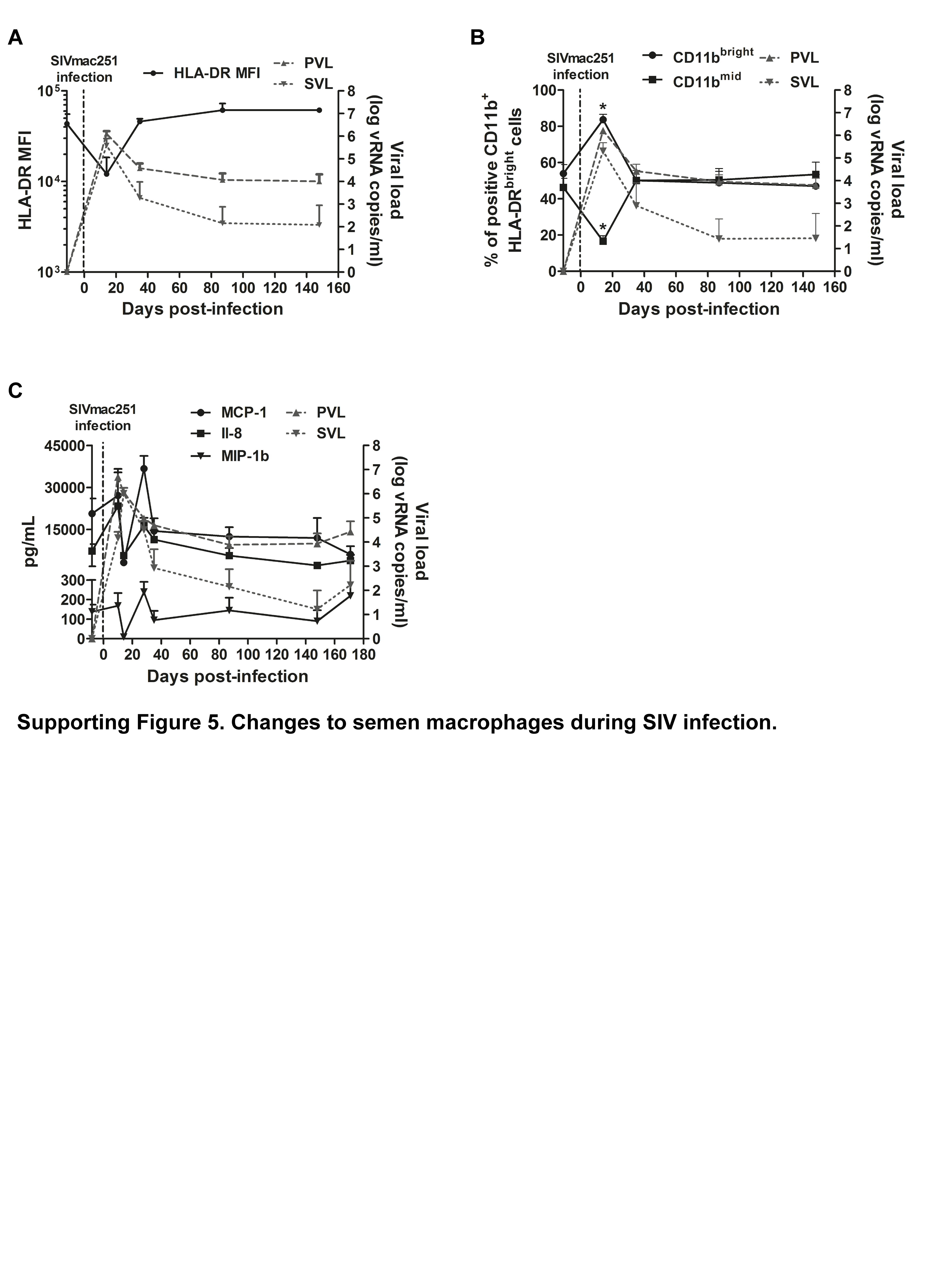

Supplement: Figure S5 — Changes to semen macrophages during SIV infection. (A) Longitudinal follow-up of the mean fluorescence intensity (MFI) for HLA-DR in infected macaques (at 14 and 35 dpi, data were available for only 4 macaques). (B) Longitudinal follow-up of the proportion of CD11bbright and mid among total CD11b+ HLA-DRbright cells (C) Dynamics of seminal plasma MCP-1, Il-8 and MIP-1b concentrations, as determined with Luminex technology. (A–C) Dotted lines represent PVL (plain triangle base dow) and SVL (plain triangle base up). (TIF) [file ppat.1003810.s005.tif]

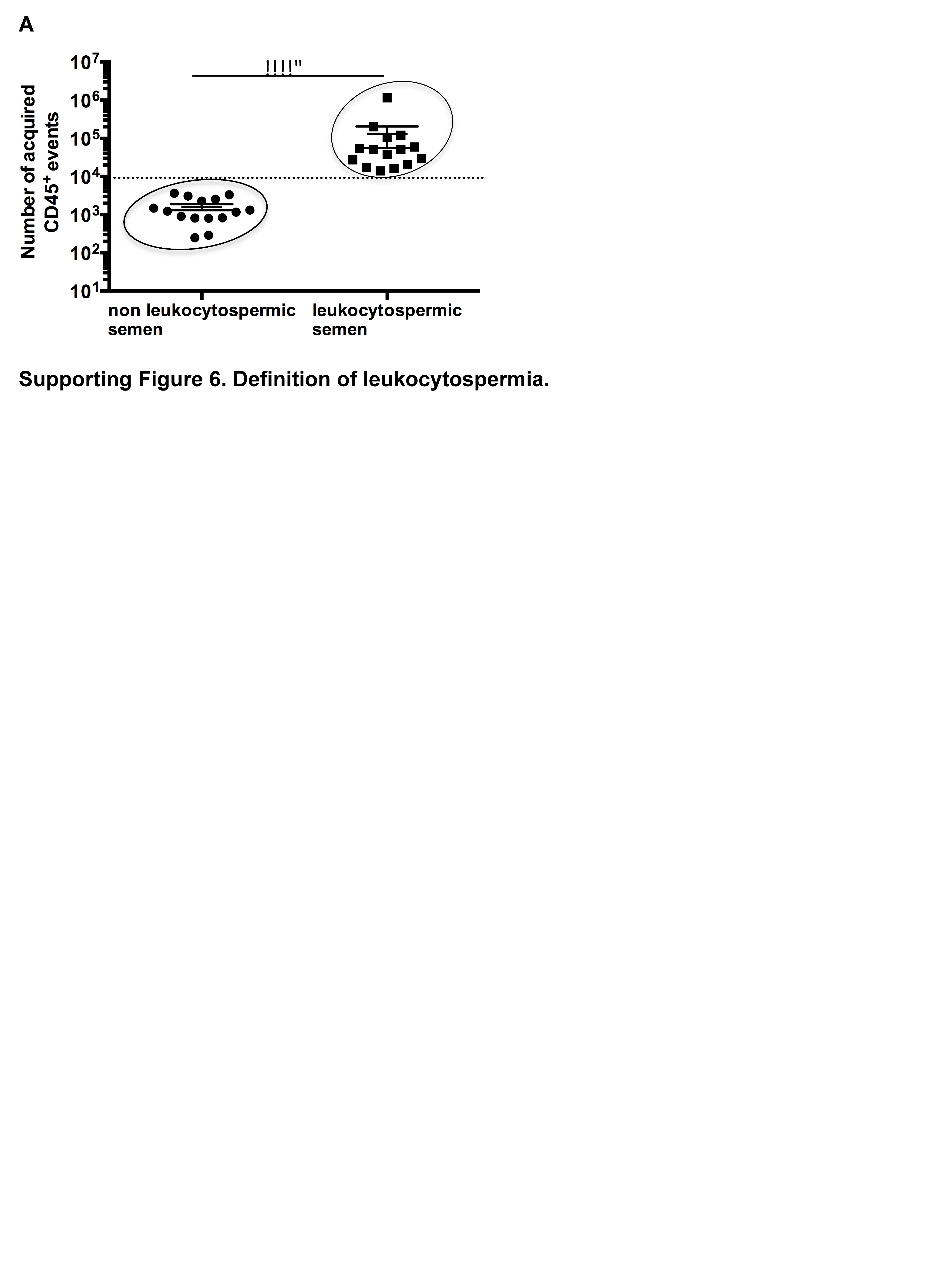

Supplement: Figure S6 — Definition of leukocytospermia. Number of total acquired CD45+ events in non-leukocytospermic and leukocytospermic macaques (n = 15 animals in each group). Mean and SEM are represented. A cut-off is defined at 10,000 positive events. (TIF) [file ppat.1003810.s006.tif]

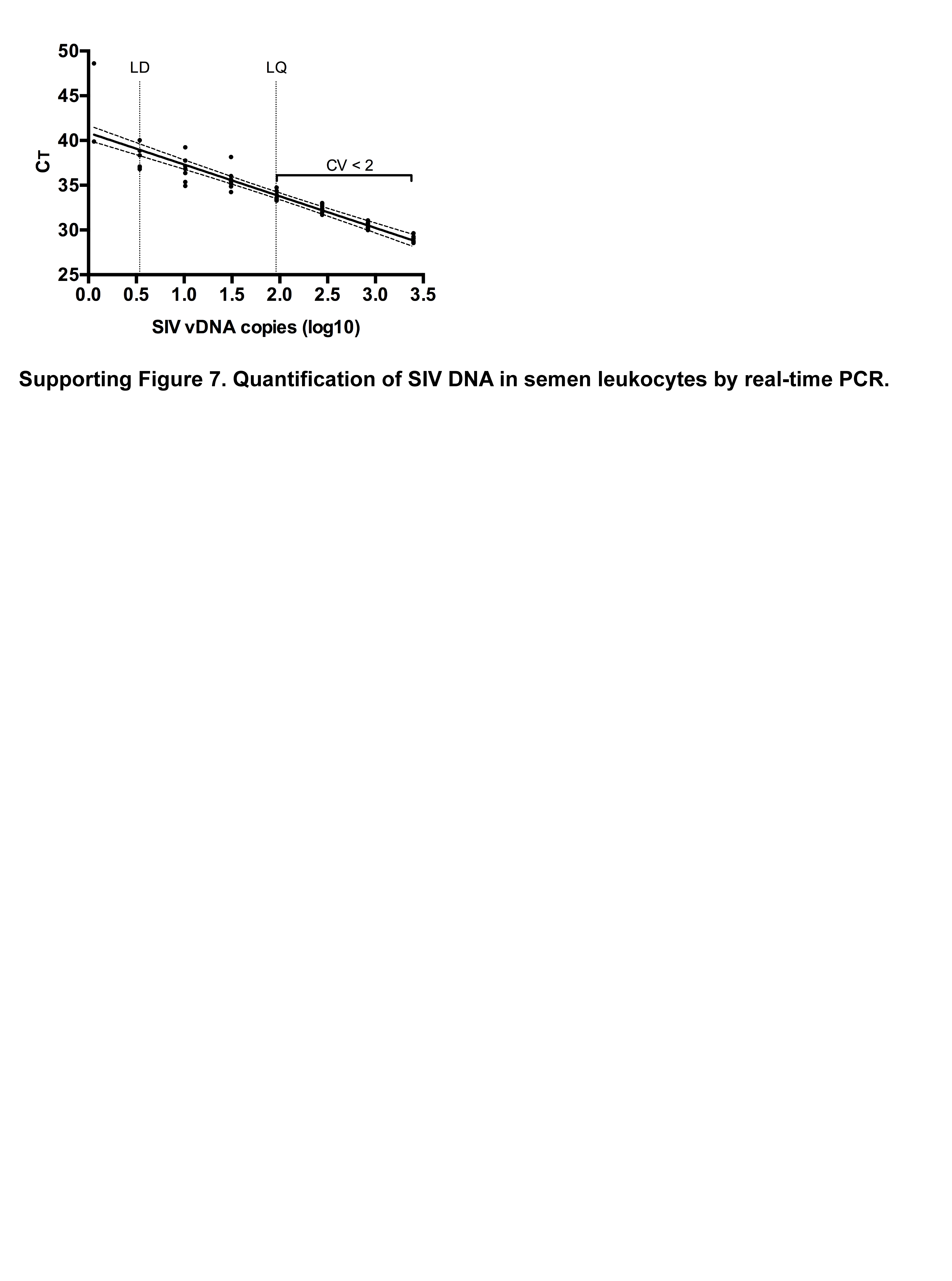

Supplement: Figure S7 — Quantification of SIV DNA in semen leukocytes by real-time PCR. Quantification of SIV DNA copies using a standard dilution of a SIV Gag plasmid diluted in macaques genomic DNA. The data of four different experiments conducted in duplicates are represented. Linear regression is represented with mean and SEM (95% Cl.). %CV: coefficient of variation as a percentage (%CV = 100*standard deviation/mean). The limit of quantification (LQ) is 90 SIV DNA copies (%CV = 1.57). The limit of detection (LD) is 3 SIV DNA copies. Background threshold is defined at 40 threshold cycle (CT, Y axis). (TIF) [file ppat.1003810.s007.tif]
