# Supplementary material for: Semen CD4+ T Cells and Macrophages Are Productively Infected at All Stages of SIV infection in Macaques
Source: PLoS Pathog. 2013 Dec 12;9(12):e1003810. doi: 10.1371/journal.ppat.1003810 (PMC3861532; doi:10.1371/journal.ppat.1003810)
Supplement: Protocol S1 — Antibodies panel for semen T cells and antigen-presenting cells phenotyping. No legend. (DOCX) [file ppat.1003810.s008.docx]

**Protocol S1. Antibodies panel for semen T cells and antigen-presenting cells phenotyping.**

| **Antibody** | **Fluorochrome** | **Clone** | **Supplier** |
| --- | --- | --- | --- |
| **Backbone (shared by panels 1-4)** | | | |
| **CD45** | PerCp | B058-1283 | BD Biosciences |
| **CD3** | V500 | SP34-2 | BD Biosciences |
| **CD4** | PE-Cy7 | L200 | BD Biosciences |
| **CD8** | V450 | BW138/80 | Miltenyi Biotec |
| **CD11b** | Alexa Fluor 700 | ICRF44 | BD Biosciences |
| **HLA-DR** | APC-H7 | G46-6 | BD Biosciences |
| **Panel 1: T cell activation and differentiation** | | | |
| **CD69** | FITC | FN50 | BD Biosciences |
| **CD95** | APC | DX2 | BD Biosciences |
| **CD28** | ECD | 25-0289-73 | Clinisciences |
| **Panel 2: Macrophages characterization** | | | |
| **CD14** | V450 | M5E2 | BD Biosciences |
| **CD66** | FITC | TET2 | Miltenyi Biotec |
| **CD163** | APC | 215927 | R&D Systems |
| **Panel 3: CCR5 and CXCR4 expression on T cells and macrophages** | | | |
| **CCR5** | APC | 3A9 | BD Biosciences |
| **CXCR4** | PE | 12G5 | BD Biosciences |
| **Panel 4: LFA-1 and Mac-1 expression on T cells and macrophages** | | | |
| **CD11a** | PE | HI111 | BD Biosciences |
| **CD18** | APC | 6.7 | BD Biosciences |
| **Panel 5: Macrophages and dendritic cells characterization** | | | |
| **CD45** | PerCp | B058-1283 | BD Biosciences |
| **CD11b** | Alexa Fluor 700 | ICRF44 | BD Biosciences |
| **HLA-DR** | APC-H7 | G46-6 | BD Biosciences |
| **CD3** | V450 | SP34-2 | BD Biosciences |
| **CD8** | V450 | BW138/80 | BD Biosciences |
| **CD20** | V450 | L27 | BD Biosciences |
| **CD141 (BDCA3)** | PE | 1A4 | BD Biosciences |
| **CD14** | FITC | M5E2 | BD Biosciences |
| **CD123** | PE-Cy7 | 7G3 | BD Biosciences |
| **CD163** | APC | 215927 | R&D Systems |
