# Supplementary material for: Semen CD4+ T Cells and Macrophages Are Productively Infected at All Stages of SIV infection in Macaques
Source: PLoS Pathog. 2013 Dec 12;9(12):e1003810. doi: 10.1371/journal.ppat.1003810 (PMC3861532; doi:10.1371/journal.ppat.1003810)
Supplement: Protocol S2 — Primers and Taqman probe specific to cynomolgus macaque CCR5 gene. Nucleotide sequence. (DOCX) [file ppat.1003810.s009.docx]

**Protocol S2. Primers and Taqman probe specific of cynomolgus macaque CCR5 gene.**

| **CCR5 gene amplification** | |
| --- | --- |
| **Forward primer** | CTG CAG CTC TCA TTT TCC A |
| **Reverse primer** | CCC GAG TAG CAG ATG ACC |
| **Probe** | ACA AGC AGC GGC AGG ACC AGC C |
