# Supplementary material for: Semen CD4+ T Cells and Macrophages Are Productively Infected at All Stages of SIV infection in Macaques
Source: PLoS Pathog. 2013 Dec 12;9(12):e1003810. doi: 10.1371/journal.ppat.1003810 (PMC3861532; doi:10.1371/journal.ppat.1003810)
Supplement: Table S1 — Influence of SIV infection on molecules more abundant in leukocytospermic semen. * : Mann-Whitney test p control versus chronic infection. Mean and SEM are specified. Normal semen group: n = 12, leukocytospermic semen group: n = 13. (DOCX) [file ppat.1003810.s010.docx]

**Table S1. Influence of SIV infection on molecules more abundant in leukocytospermic semen.**

| **Molecule** | **Uninfected macaques (pg/ml)** | | **Mann-Whitney test *p* control *versus* primary infection** | **Correlation with PVL** | | **Correlation with SVL** | |
| --- | --- | --- | --- | --- | --- | --- | --- |
|  | **Normal semen** | **Leukocyto-spermia** |  | **Spearman correlation *p*** | **Spearman correlation *r*** | **Spearman correlation *p*** | **Spearman correlation *r*** |
| **MCP-1** | 3,646 ± 1,020 | 26,775 ± 4,762 | 0.2475******* | 0.0099 | 0.5376 | 0.5820 | 0.1211 |
| **IL-8** | 548.00 ± 144.30 | 7,838 ± 1,997 | 0.0027 | <0.0001 | 0.7445 | <0.0001 | 0.7494 |
| **RANTES** | 94.29 ± 25.34 | 355.00 ± 49.12 | 0.0007 | 0.0004 | 0.6857 | 0.0023 | 0.6031 |
| **IL-6** | 14.69 ± 4.13 | 60.20 ± 11.80 | 0.1765 | 0.0157 | 0.5082 | 0.0033 | 0.5854 |
| **IP-10** | 0.36 ± 036 | 15.58 ± 5.412 | 0.0108 | 0.144 | -0.5046 | 0.0347 | -0.6809 |
